# Different effects of verbal and visual working memory loads on Language prediction

**DOI:** 10.1038/s41598-025-03556-w

**Published:** 2025-07-01

**Authors:** Shun Liu, Wenpeng Hu, Xiqin Liu

**Affiliations:** 1 Department of Education, Hunan First Normal University, Changsha, China; 2 School of Foreign Languages, South China University of Technology, 381 Wushan Road, Guangzhou, 510641, China; 3 Department of Psychology, University of Jyvaskyla, Jyväskylä, Finland; 4 Department of Theoretical and Applied Linguistics, University of Cambridge, Cambridge, UK; 5 Department of Student Affairs, Guangdong Songshan Polytechnic, Shaoguan, China

**Keywords:** Visual-spatial working memory load, Verbal working memory load, Language prediction, Tonal prediction, Visual world paradigm, Psychology, Communication and replication

## Abstract

Mounting studies suggest that working memory (WM) plays a crucial role in language prediction, but how varying types of WM loads influence language prediction remains unclear. This study investigated whether verbal and visual WM loads differentially impact language predictions during speech comprehension. Using a dual-task paradigm combined with eye-tracking in a visual world setting, we asked 48 participants to complete a sentence comprehension task under concurrent WM load conditions. Participants were divided into two groups, one of which performed a visual dots memory task and the other completed a visual words memory task, with memory load being applied in half of the trials. Results revealed anticipatory gaze towards target objects, suggesting the prediction of upcoming linguistic information. Notably, early fixations during the tonal cue window indicated tonal prediction in spoken sentence processing. Furthermore, WM load significantly disrupted participants’ language prediction effects, highlighting the involvement of working memory resources in this process. Importantly, the verbal memory task imposed a more severe disruption to language prediction than the visual memory task, suggesting differential roles of WM subtypes in linguistic prediction. This offers novel insights into how verbal WM and visual-spatial WM differentially influence predictive language processing.

## Introduction

Prediction has been posited as a crucial mechanism for managing the fleeting nature of memory during rapid language comprehension^1^. Listeners can generate the corresponding representations in advance of subsequent input. By pre-activating upcoming linguistic information, the brain reduces the cognitive demands of real-time processing, thereby facilitating efficient communication^2^. Despite its role in mitigating memory limitations, predictive processing depends on working memory (WM) resources to retain and manipulate the contextual information necessary for accurate predictions. While several studies have suggested the influence of WM resources on predictive language processing^3–5^, the impact of WM load on language predictions across different modalities remains less understood. To address this gap, the current study employed a dual-task paradigm with eye-tracking in a visual world setting to examine how verbal and visual WM loads differentially impact language prediction during speech comprehension.

The strong prediction view claims that predictive processing operates across all levels of linguistic information^6,7^. According to this viewpoint, the brain actively generates predictions about upcoming linguistic elements, ranging from low-level phonological cues to high-level semantic meaning. For instance, people can predict phonological elements, such as the initial segments^8^, and semantic meanings^9^. However, the degree and level of predictive processing might depend on various factors, including cognitive resource constraints^2,10^.

There is evidence that individuals’ WM capacity and WM load significantly affect their predictive performance during language comprehension. For instance, Huettig and Janse identified the influence of WM capacity on gender prediction using the visual world paradigm (VWP)^3^. In this study, Dutch native speakers listened to spoken instructions (e.g., “Kijk naar de_COM_ afgebeelde piano_COM_” – look at the displayed piano, or “Kijk naar het_NEU_ afgebeelde paard_NEU_” – look at the displayed horse) while viewing four objects. The ‘_COM_’ and ‘_NEU_’ markers denote genders. The gender-concordant articles (het for neuter, de for common) provided cues for predicting the upcoming noun. Anticipatory eye movements toward the gender-matched object before the target word was spoken indicated successful prediction of the noun’s gender. Furthermore, by measuring participants’ general WM capacity—including verbal, numerical, and visual-spatial domains—the researchers found that higher WM capacity correlated with stronger anticipatory fixation effects. This finding underscores the pivotal role of WM in supporting predictive language processing^11^.

More relevantly, Ito et al. investigated the effect of verbal WM load on anticipatory fixations using a dual-task paradigm combined with eye-tracking in a visual world setting^4^. Participants engaged in a language comprehension task while simultaneously performing a verbal WM task that required memorizing words. The study found that verbal WM load delayed participants’ anticipatory eye movements to target objects, indicating that verbal WM is essential for semantic prediction. Similarly, Li and Qu examined the influence of verbal WM capacity on semantic and phonological predictions^5^. Their findings revealed that participants with higher verbal WM capacity exhibited earlier anticipatory eye movements than those with lower capacity during semantic prediction conditions.

While these studies highlight the critical role of WM in predictive language processing, little is known about how different types of WM load—such as verbal versus visual-spatial—affect language prediction. The multi-component model of WM^12,13^ provides a useful framework for addressing this gap. According to this model, verbal WM and visual WM rely on distinct cognitive resources, which may differ in their contribution to language comprehension. Verbal WM, involving phonological storage and rehearsal, is closely tied to linguistic processing by supporting the retention and manipulation of phonological information. Consequently, verbal WM may play a more pivotal role in tasks requiring phonological and semantic predictions.

In contrast, the role of visual WM in language prediction remains less well understood, but emerging evidence suggests that it may influence language comprehension through situational modeling and contextual integration^14^. For example, visual WM has been implicated in maintaining spatial representations of discourse settings and tracking referential relationships in conversation^15,16^. In other words, visual WM allows us to create and maintain these mental “maps” of the discourse world, helping us to understand the relationships between characters, objects, and events as they unfold in the described setting. Additionally, studies have shown that prediction in language processing is not limited to verbal cues but can also be shaped by visual and spatial context^17,18^. These distinct cognitive demands suggest that verbal WM may have a direct influence on language prediction, whereas the role of visual WM might be less specific or secondary.

To investigate whether verbal and visual WM loads differentially would impact language prediction during speech comprehension, we adapted a dual-task paradigm with eye-tracking in a visual world setting. The dual-task WM and sentence comprehension paradigm is widely used to manipulate comprehenders’ WM load during language processing^4,19,20^. By asking comprehenders to remember the verbal or nonverbal material while completing a sentence comprehension task, this paradigm allows for targeted manipulation of the type of WM load imposed during comprehension.

To construct predictive contexts, we employed the tone sandhi and classifier systems in Chinese speech to elicit language predictions. Tone sandhi refers to tonal alternations in tonal languages, such as Chinese, where a word’s tone changes depending on the tone of the subsequent word, while other linguistic features remain constant^21^. For example, the word “一” (“one”) takes a rising tone, [yi2], in the phrase “一辆单车” (“a bike”), but shifts to a falling tone, [yi4], in the phrase “一张邮票” (“a stamp”). Here, the numbers following “yi” (i.e., “yi2” and “yi4”) indicate the tone of the Mandarin Chinese syllable, following the IPA (International Phonetic Alphabet) standard for tone marking in Pinyin transcription. This phenomenon offers a unique opportunity to investigate the pre-activation of tonal information while minimizing confounding factors, such as semantic or orthographic influences. Supporting the use of tone sandhi in prediction research, Ito and Hirose^22^ demonstrated that predictable tones in Japanese tone sandhi facilitated faster responses in recognition tasks, providing evidence for phonological prediction.

Chinese classifiers, which are used before nouns modified by numbers (e.g., one, two), offer another predictive cue^23^. Classifier usage is governed by the semantic features of the noun^24,25^. For example, in the phrase “一辆单车” (“a bike”), the classifier “辆” is used for “单车” (“bike”), as “辆” is the classifier for vehicles like a bike, and in the phrase “一张邮票” (“a stamp”), the classifier “张” is used for “邮票” (“stamp”), as “张” is the classifier for flat objects like a stamp. This characteristic of the classifier allows comprehenders to infer the semantic features of its following noun, and previous studies have provided language prediction evidence based on Chinese classifiers^26,27^.

To measure language prediction, we employed an eye-tracking visual world paradigm. Participants listened to sentences such as “请问有一[yi4]张邮票在右边吗?” (“Is there a stamp on the right?”) while viewing images containing two objects: a stamp and a bike. The tone sandhi cue “一[yi4]” matched the tone of the classifier “张”, which is used for “邮票” (“stamp”), but not the classifier “辆”, which is used for “单车” (“bike”). Anticipatory fixations on the stamp before the word “邮票” was presented served as an indicator of pre-activation of the word “邮票”.

Building on Fennell et al.’s^20^ approach to manipulating WM load, we imposed additional verbal or visual WM tasks on half of the trials. Participants in the verbal WM condition memorized words, while those in the visual WM condition memorized spatial dot patterns. We then compared anticipatory fixations during the predictive time window when tone sandhi and classifier cues were presented. We hypothesized that: (1) participants would fixate more on the predictable target object during the predictive time window; (2) WM load would disrupt anticipatory fixations; and (3) verbal WM load would exert a greater disruptive effect than visual WM load.

## Methods

### Participants

We recruited 24 native speakers of Mandarin Chinese for each group. All participants had normal or corrected-to-normal vision and no history of language disorders. Each of them provided informed consent prior to the study. Participants in the visual WM group (16 females) had a mean age of 21.0 years (SD = 2.71 years), while those in the verbal WM group (15 females) had a mean age of 20.6 years (SD = 2.34 years). All of them were compensated for their participation. The study protocol was reviewed and approved by the Ethics Committee of the South China Normal University [No. SCNU-PSY-2021-082]. All experiments were performed in accordance with relevant guidelines and regulations.

## Materials and design

This study employed a mixed design with two within-subject conditions (load vs. no-load) and two between-subject conditions (visual WM load vs. verbal WM load). The experiment was divided into two blocks: a dual-task block and a single-task block. In the dual-task block, participants completed 56 trials that required them to remember a WM stimulus while performing an eye-tracking visual world task. In the single-task block, participants completed 56 trials that only involved the eye-tracking task. In the visual WM group, the stimuli consisted of visuospatial dot layouts, as used in Fennell et al.’s^20^ study. In the verbal WM group, the stimuli were three Chinese nouns, adapting the design from Fennell et al. The order of the two blocks was counterbalanced between participants in each group to mitigate potential order effects.

**Fig. 1:**
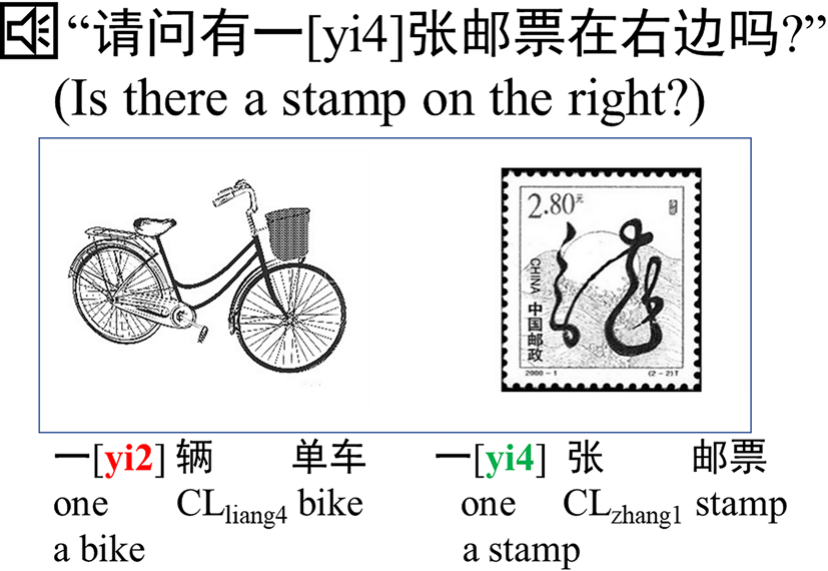
A sample visual display with the auditory sentence for the eye-tracking visual world task. The tone sandhi of the number for the target object differs from that for the competitor (e.g., target: *“一[yi4]张邮票”*, competitor: *“一[yi2]辆单车”*). Consequently, the tone sandhi serves as a predictive cue for inferring the classifier, which can hint at the target object via the classifier’s semantics. When the classifier is presented, the target noun can be pre-activated more directly via the semantic link between the classifier and the target object.

The stimuli for the eye-tracking visual world task comprised spoken sentences paired with black-and-white line drawings (see Fig. 1). To address the potentially weaker predictive effects of tone sandhi, the experiment used a simplified two-object visual scene instead of a more complex four-object design. In this task, participants utilized the tone of the numeral “一[yi4]” to predict the tone of the subsequent classifier, which, in turn, cued the target object through the classifier’s semantic properties. For instance, the classifier “张[zhang1]” (used for flat objects like a stamp) aligns tonally with “一[yi4]”, making “一[yi4]张邮票” an appropriate combination. Conversely, “辆[liang4]” (used for vehicles like a bike) does not match “一[yi4]”, making “一[yi4]辆单车” inappropriate. This tonal alignment enabled the numeral “一[yi4]” to serve as a predictive cue for fixation patterns toward the target (stamp) versus the competitor (bike). Additionally, when the classifier was presented, the semantic association between the classifier and the target object further facilitated the pre-activation of the noun.

To ensure the classifiers were highly specific to their associated objects, we conducted a norming test using a cloze task^28^ with 15 Mandarin speakers. These participants did not participate in the eye-tracking experiment. Participants were shown an object and a phrase fragment with the classifier omitted (e.g., “一___邮票”, meaning “one ___ of stamp”). Objects for which more than 85% of participants provided the same classifier were included, resulting in a final selection of 112 objects. Half of these objects were paired with classifiers associated with [yi4], and the other half with classifiers associated with [yi2].

We constructed 112 spoken sentences, recorded by a female native Mandarin speaker at a 48 kHz sampling rate. Each sentence included the following segments: a common introductory phrase (“Is there. on the.”), a tone sandhi numeral ([yi4] or [yi2]), a classifier, a noun, and a location word (“right” or “left”). Half of the sentences included [yi4], and the other half included [yi2]. For each tone sandhi condition, half of the sentences used “right” as the location word, and half used “left”. These segments were carefully recorded and assembled into complete sentences (e.g., “请问有一张邮票在右边吗?”, meaning “Is there a stamp on the right?”). The syllable onset asynchrony was set to 800 ms, following prior studies that identified word-form prediction effects at 700 ms but not at 500 ms^29^. The additional delay aimed to ensure phonological prediction effects could fully manifest within the time window.

Each sentence was paired with two visual arrangements to create two scenarios, with the target object appearing on the right of the screen (e.g., a bike and a stamp) or on the left (e.g., a stamp and a bike). Coupled with each item’s appearance in either the load or no-load block, this design yielded four stimulus lists. These lists were counterbalanced across participants to control for position and item effects on fixation patterns.

### Individual differences measures

#### Verbal working memory: Nonword repetition

An auditory nonword repetition (NWR) task was used to assess verbal/phonological working memory^30^. Participants completed the task in a sound-attenuating booth, where they heard 48 phonotactically legal Chinese nonwords presented through headphones at a fixed level of 70 dB SPL. Each nonword was played once, after which participants repeated it. Inter-trial intervals were three seconds. The nonwords varied in length from two to five syllables and were presented in a fixed order for all participants^3^. Performance was measured by the number of phonologically repeated nonwords, with higher scores indicating better auditory verbal working memory. A two-sample t-test comparing the visual WM group to the verbal WM group showed no significant difference in verbal working memory capacity between the two groups (t = 0.32, *p* =.75).

#### Spatial working memory: Corsi block task

The visual Corsi block tapping task^31^ was used to assess visuospatial working memory^3^. In this computerized variant, participants viewed nine identical blocks arranged irregularly on a screen. A subset of these blocks was sequentially highlighted at a rate of one per second, and participants were required to replicate the sequence by clicking the corresponding blocks in the same order. The task progressively increased in difficulty as sequence lengths ranged from 2 to 9, with two trials per length, totaling 16 trials. Performance was measured by the number of accurately reproduced sequences, with higher scores indicating better visuospatial working memory. A two-sample t-test comparing the visual WM group and the verbal WM group showed no significant difference in visual working memory capacity between the two groups (t = 1.49, *p* =.14).

### Apparatus and procedure

Prior to the experiment, participants were instructed to memorize the names of all objects without their associated classifiers—for instance, “邮票” (“stamp”) rather than “一张邮票” (“a stamp”). This memorization phase ensured that participants predicted the appropriate classifier during the experiment rather than simply recalling it from memory. The formal experiment commenced only after participants successfully demonstrated recognition of all objects used in the study.

Eye movements were recorded using an EyeLink 1000 Tower-Mount eye tracker (SR Research Ltd., Osgoode, Ontario, Canada) at a sampling rate of 500 Hz. Experimental materials were displayed on a 21-inch CRT monitor with a resolution of 1280 × 1024 pixels. Before the experiment began, a 13-point calibration was performed to ensure accuracy. Participants sat at a distance of 60 cm from the visual display and were instructed to look at the pictures while listening to the sentences, during which their eye movements were recorded. Three practice trials were conducted before the formal experiment to familiarize participants with the task.

**Fig. 2:**
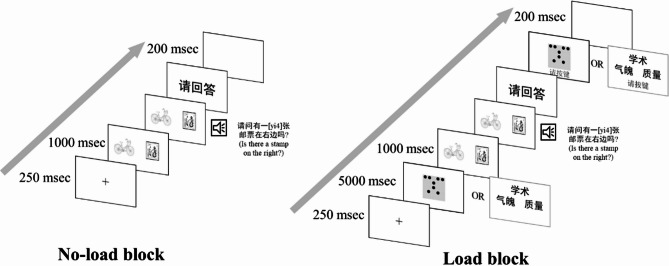
Trial procedures. The left panel shows the no-load block and the right panel shows the load block. In the load block, the memory task could be a dots memory task or a word memory task.

In the no-load block, each trial began with a drift check followed by the presentation of a 250-ms fixation cross. Visual objects were displayed 1000 ms prior to the onset of the auditory sentence and remained on the screen until the sentence ended. In one-third of the trials, participants saw a prompt (“Please answer”) after the sentence and were required to respond with “yes” or “no” aloud. This behavioral task—judging whether the spoken sentence matched the visual scene—served primarily as a means to ensure participants remained attentive to both the visual and auditory stimuli throughout the experiment. Without an overt task, there is a risk that participants might not engage fully with the materials, which could diminish the sensitivity to detect prediction-related effects. These behavioral responses were not treated as an independent or dependent variable in our analyses. The response trials were randomly distributed, and participants were only cued to respond after hearing the sentence. Therefore, they had to pay close attention in every trial to ensure accurate performance when a response was required. This approach allowed us to maintain participant engagement without requiring a response on every trial, which helped reduce the total experiment duration and minimize participant fatigue. Following their response, participants pressed the space bar to proceed to a 200-ms blank screen (see Fig. 2 left panel).

The load blocks were nearly identical to the no-load block, except that participants were additionally required to complete a WM task (see Fig. 2 right panel). Specifically, for the visual WM group, participants were shown a dot matrix for 5 s prior to the language prediction stimuli. This image contained spatially organized dots and was designed to occupy the participants’ visuospatial WM resources^20^. After the language prediction part, participants viewed a second dot matrix and judged whether it was identical to the one presented earlier.

For the verbal WM group, participants were asked to memorize three words for 5 s before the onset of the language prediction stimuli. This task was intended to tax verbal WM resources^4,20^. Following the prediction part, participants were presented with three words and asked to judge whether the words matched those presented earlier.

The experiment took approximately 45 min for each participant.

### Statistical analysis

**Fig. 3:**
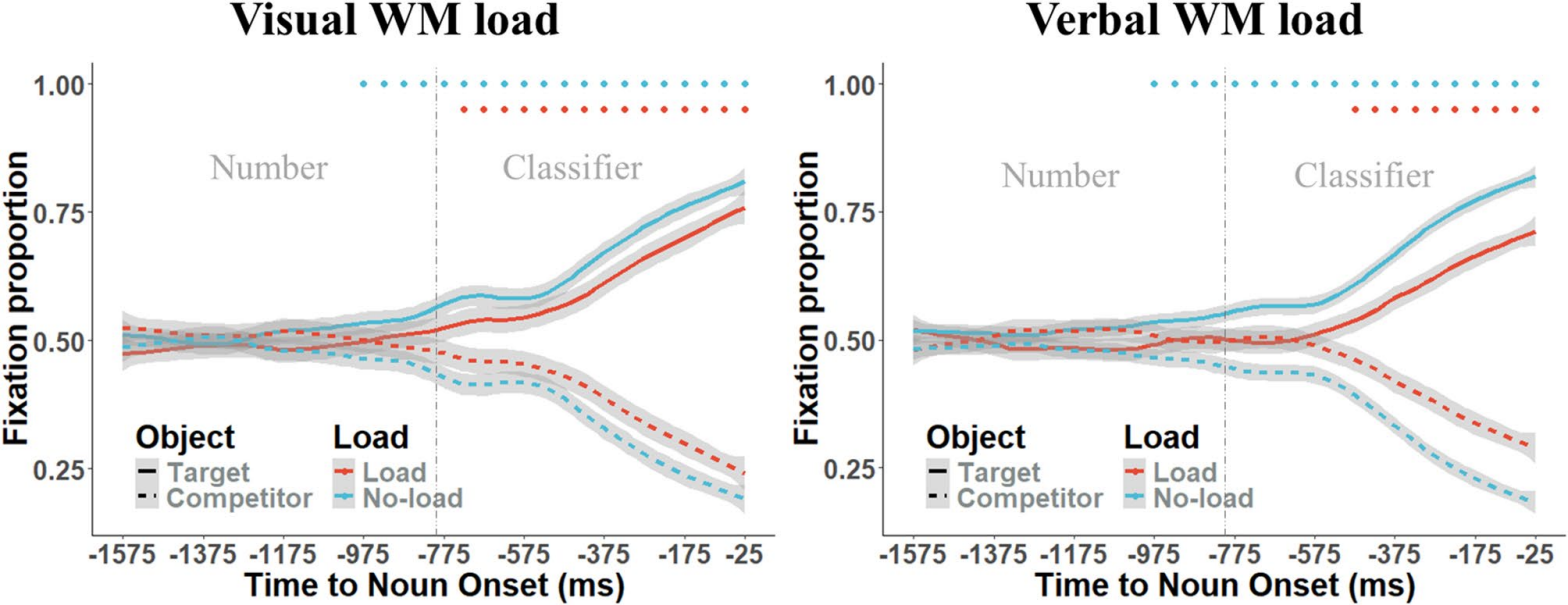
Proportions of fixations on the targets and competitors in two participant groups. The grey shadow represents the 95% confidence interval, providing a visual representation of the level of confidence in our data. The vertical grey dashed line marks the exact onset of the classifier within the timeline. To highlight the periods of particular significance, colour-coded dots are positioned at the top of the figure. These dots indicate the time windows where clusters of data reached statistical significance, helping us identify critical points in our analysis.

We analyzed the fixations on target and competitor images within the visual displays, focusing on the proportions of fixations in each 50-ms bin from 1600 ms before the onset of the spoken target noun until the noun onset (see Fig. 3). This time period is referred to as the “predictive window”. To assess fixation proportions, we used a Bayesian logistic mixed-effects model with the *brms* package^32^. Compared to traditional frequentist methods, Bayesian mixed-effects model helps mitigate model fitting issues^33^. We applied this model to each 50-ms bin within the predictive window to explore the time course of effects. Fixations were coded binomially, with a value of 1 assigned if the participant fixated on the relevant object within the 50-ms time bin, and 0 otherwise. We then compared fixations on the target versus the competitor to examine prediction effects in each condition. A uniform model was constructed, incorporating by-participant random intercepts and slopes for the fixed factors, as well as by-item random intercepts, which was based on comprehensive consideration of model scientific, model uniformity, model fitting, and model maximization^34^. To address the multiple comparisons problem in the predictive window, we conducted cluster-based permutation tests on the fixation proportions using the *permuco* package^35^.

Second, to directly compare the effects of different WM loads on predictive fixations, we conducted growth curve analyses to examine how the load presence (no-load vs. load) and load type (visual vs. verbal WM load) affected the cumulative rate of fixations on the target relative to the competitor during the predictive window^36^. A cubic orthogonal polynomial of time was included in the growth curve analysis to account for potential nonlinear dynamics in fixations over time. This approach, commonly used in growth curve modeling^37,38^, ensures that the estimate of the linear term (hereafter referred to as time1), which is the primary focus of our analysis, is not confounded by higher-order trends. We used a Bayesian linear mixed-effects model (LMM) with the *brms* package^32^, constructing a uniform model that included by-participant random intercepts and slopes for both load presence and load type, as well as by-item random intercepts.

Participants’ accuracy in the comprehension task was analyzed using a Bayesian logistic mixed-effects model with the *brms* package^32^. This model included by-participant random intercepts and slopes for the fixed factors, as well as by-item random intercepts. The data and materials for all experiments are available at https://osf.io/58q9b/?view_only=fe9f7f33a08d4f72811ae1efd5d1ac1b, and none of the experiments was preregistered.

## Results

The presence of load and load type did not affect participants’ accuracy in the comprehension task. The Bayesian logistic mixed-effects model analysis showed that the load effect was − 0.2 (95%CI = [−1.4, 1.1]), the group effect was 0.3 (95%CI = [−0.6, 1.2]), and the interaction between load and group is −0.3 (95%CI = [−1.2, 0.6]). Figure 3 shows the proportion of fixations on the target or competitor object from the onset of the number to the onset of the noun. In the no-load condition, both groups fixated more on the target objects during the time window of the number. The bin analysis revealed that both groups fixated more on the target starting 175 ms before the onset of the classifier. Cluster-based permutation tests confirmed the significance of these clusters (*mass*_*visual*_ = 1953, *p* <.001; *mass*_*verbal*_ = 1286, *p* <.001).

In contrast, in the load condition, the visual group fixated more on the target objects starting 75 ms after the onset of the classifier, while the verbal group fixated more on the target starting 325 ms after the classifier onset. Cluster-based permutation tests confirmed the significance of these clusters (*mass*_*visual*_ = 858, *p* <.001; *mass*_*verbal*_ = 447, *p* <.001).

**Fig. 4:**
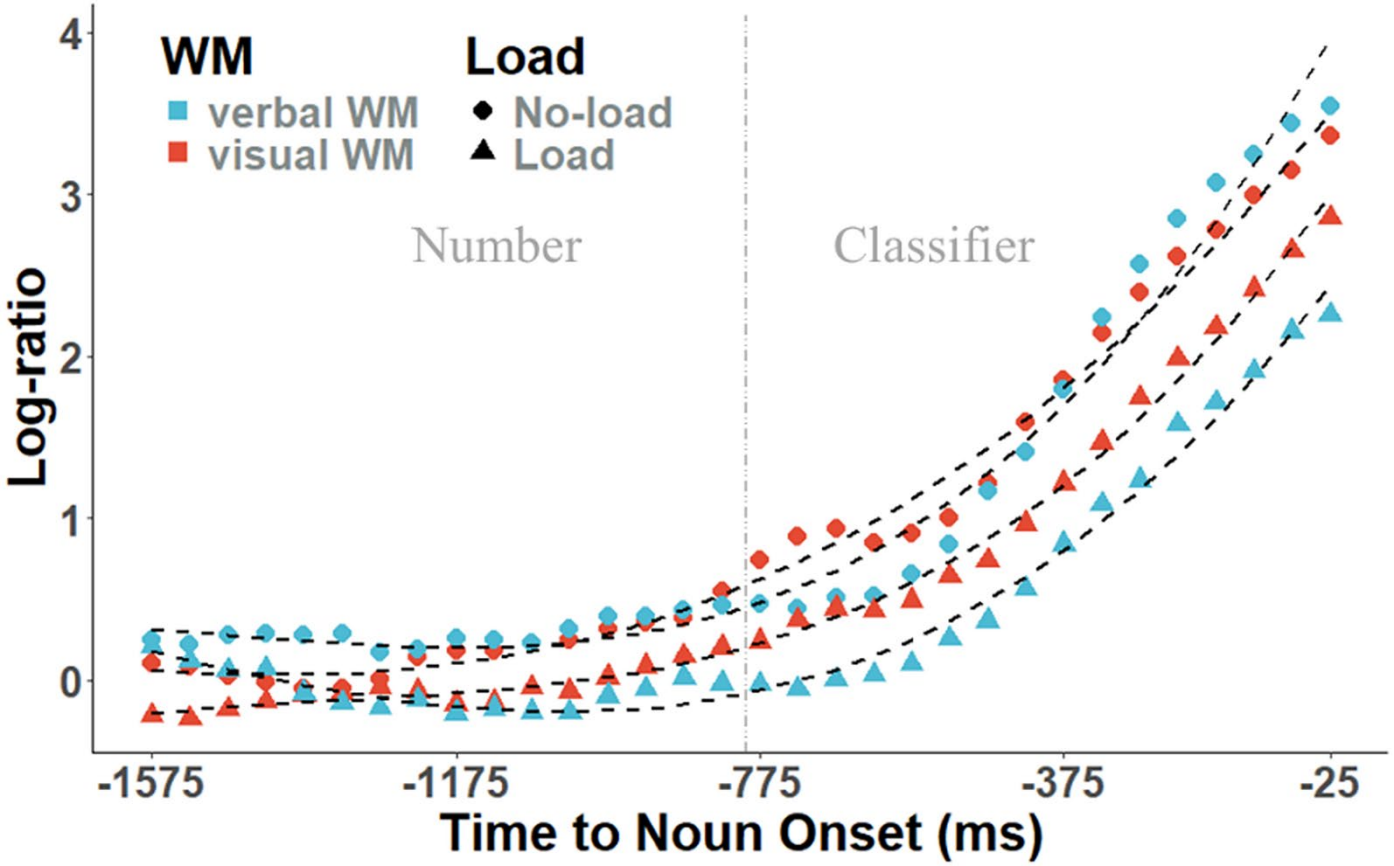
Growth curve analysis in the predictive window. The points represent the average log-ratio scale, while the curves illustrate the model fits. The color of points indicates the type of WM load, visual or verbal, while the shape (triangle or diamond) indicates load or no-load conditions. The vertical grey dashed line marks the precise onset of the classifier within the timeline.

**Fig. 5:**
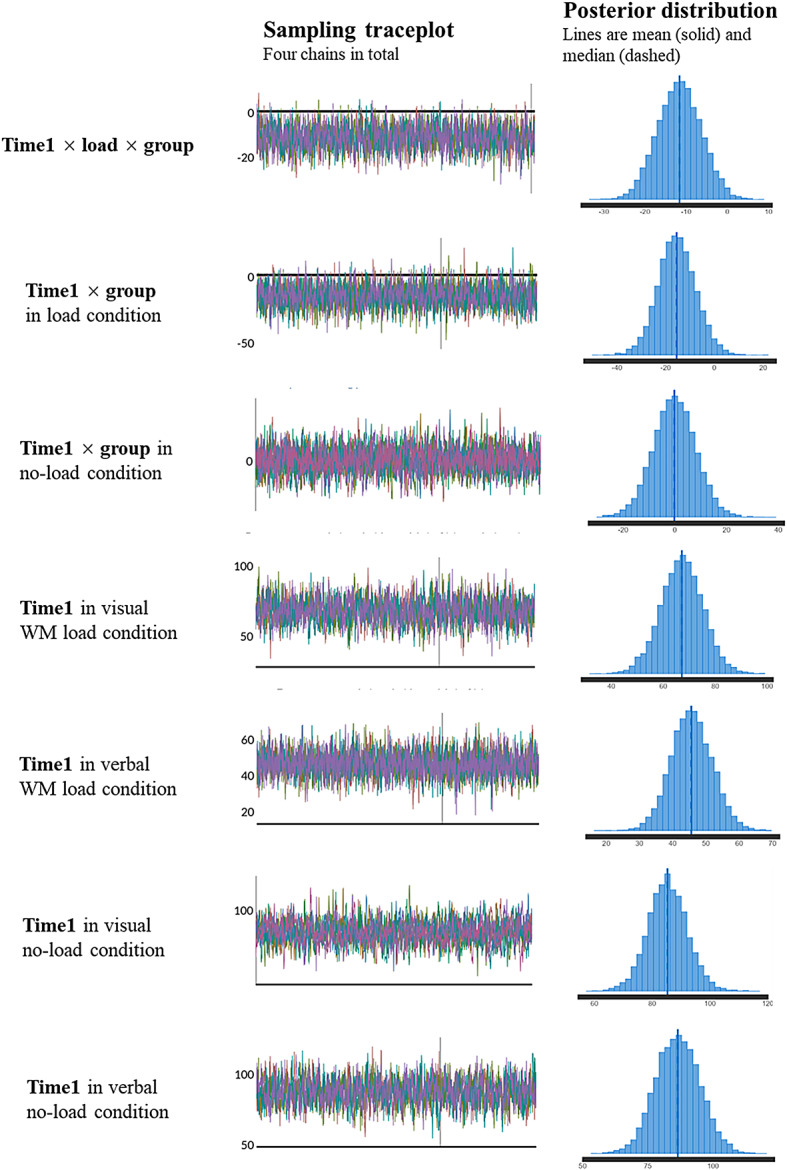
Bayesian LMM analysis main results. The left column shows the effect names, the middle column shows the traceplot of model fitting, and the right column shows the posterior distribution of each effect. The sampling traceplot is primarily interpreted visually to assess the convergence and mixing of Markov Chain Monte Carlo (MCMC) samples. The results appeared as well-mixed, stationary patterns without noticeable trends or drifts, indicating good convergence and efficient sampling.

Figure 4 illustrates the growth curve from the number onset to the noun onset. The Bayesian linear mixed model analysis (see Fig. 5 for main results) revealed a time1 × load × group interaction effect (mean_time1×load×group_ = −11.6, 95%CI = [−21.9, −1.3]). Subsequent simple effects analysis showed a larger time1 × group interaction effect in the load condition (mean_time1×group_ = −15.3, 95%CI = [−30.7, −0.4]) than in the no-load condition (mean_time1×group_ = −0.1, 95%CI = [−16.7, 16.2]). Specifically, the visual group showed a larger time1 effect than the verbal group in the load condition (mean_visual_ = 67.3, 95%CI = [50.9, 82.8]; mean_verbal_ = 45.7, 95%CI = [33.5, 58.2]), while an almost same time1 effect with the verbal group in the no-load condition (mean_visual_ = 85.3, 95%CI = [71.7, 99.1]; mean_verbal_ = 86.5, 95%CI = [70.1, 103.3]).

## Discussion

The primary goal of this study was to investigate whether and how verbal and visual WM loads differentially impact language predictions. We found that listeners fixated more on the predictable target object before they heard the target noun, which suggests that they did predict the upcoming referent. However, the delayed fixations under WM load conditions suggest that additional WM tasks disrupted this anticipatory fixation. Furthermore, we observed that the visual group fixated more on the target object starting 75 ms after the onset of the classifier, while the verbal group fixated more on the target object starting 325 ms after the onset of the classifier. The growth curve analysis also revealed that verbal WM load attenuated the growth rate of anticipatory fixations more than visual WM load did. These findings suggest that WM load affects predictive fixation during speech comprehension, with a component-specific influence.

### Language prediction

The current study reveals that participants predicted upcoming linguistic information during speech comprehension. First, listeners appear to predict tonal features of upcoming words based on tonal cues. This finding supports the “strong prediction view”^6,7^. In our experiment, when participants heard the number “一” (“one”), they fixated more on the target object than the competitor in the no-load condition. Since the only available clue for prediction at this time was the tone—and the tone could only predict the subsequent tone—we conclude that participants predicted the upcoming tone based on the tone of the number word, and then matched this pre-activated tone with the tone of the classifier for the target object.

While the effect of phonological prediction is admittedly weaker than higher-level prediction^5,38,39^, and some studies have failed to demonstrate phonological prediction effects^40^, growing evidence supports phonological prediction, particularly in VWP^5,37,38,41^. A plausible explanation is that participants are listening to auditory sentences in the VWP, where phonological information is more directly relevant.

Second, we find that listeners fixated more on the target object than the competitor during the classifier time window and this may suggest prediction of the semantic features of the upcoming noun. These results align with those of Chow and Chen^26^ who claimed that listeners exploit the semantic features of classifiers to anticipate upcoming linguistic input. Specifically, listeners exhibited anticipatory fixations on the target object following the auditory presentation of the classifier. Additionally, our data showed that predictive fixations during the classifier window were stronger than those during the number window. A potential reason for this result is that semantic predictions are generally more robust than phonological predictions^40^, or predictions based on the classifier were more direct than those based on the number’s tone. However, given that classifiers follow immediately after the tone sandhi carrier, some portion of the prediction effect observed at the classifier may be a continuation of the phonological prediction triggered by the tone sandhi. Consequently, it may also result from the accumulation of both tone and classifier cues.

However, we acknowledge that in our study, the classifier always appeared after the numeral, which introduces a potential confound with time. Even if the classifier provided no predictive cue, the effect of predictability might have increased over time simply because prediction tends to be more robust when comprehenders have more time to process the input. Future research could further disentangle these effects by varying the timing of predictive cues.

### Effects of visual and verbal WM loads on Language prediction

We found that both visual and verbal WM loads interfered with participants’ predictive eye movements to some extent. This suggests that WM resources are required to make predictive eye movements across both groups. This finding resonates with the broader claim that language prediction requires cognitive resources^2^. Note that, due to the inherent complexity of the cognitive processes engaged in memory tasks, it is difficult to attribute the observed effects solely to working memory load. This is because working memory tasks inevitably also engage more general cognitive processes, such as attention. For the sake of interpretative clarity, however, our subsequent discussion focuses on working memory as the primary factor manipulated. The effect of load indicates that participants had to allocate additional resources to the WM task in the WM load condition, thereby limiting the resources available for prediction. In contrast, participants could focus more directly on prediction in the no-load condition. Our results are consistent with existing literature^3,37^, which showed that predictive fixations are less frequent when fewer resources are available for prediction.

However, in WM load conditions, particularly verbal WM load conditions, the disappearance of predictive fixations during the number window indicates that phonological prediction was disrupted by WM load. This finding is somewhat different from the non-significant effect of verbal WM capacity on phonological prediction reported in Li and Qu’s study^5^. This discrepancy may arise from differences in experimental design: in the current study, phonological predictions were driven by phonological cues (specifically, the tone of the number), while Li and Qu’s study relied on higher-level semantics to pre-activate phonological features of specific words. The divergence in prediction pathways may be a key factor in the observed differences between the two studies. Predictions for specific words based on semantics are likely to be more robust, while phonological predictions driven by phonological cues are more susceptible to disruption due to the occupation of the phonological loop by WM load^12,13^.

It is still unclear which components of predictive fixations are most affected by WM load. In our study, comprehenders needed to identify objects in the visual scene, comprehend the speech, predict the linguistic information, judge which object matched this prediction, and then move their gaze to that object. WM load could primarily interfere with visual processes, such as identifying objects in the scene or moving the eyes to predictable objects. This is consistent with evidence suggesting that short-term memory is responsible for storing temporary spatial information^12^. Empirical evidence from Walter and Bex^42^ also indicates that cognitive load affects eye movements.

WM load may also interfere with purely linguistic prediction (i.e., predicting subsequent linguistic information) or with processes that integrate such predictions with the visual scene (e.g., determining which object matches this prediction). One possible explanation for the former is based on the prediction-by-production n^44–46^, which proposes that linguistic prediction involves an internal simulation of speech production. Given that WM load can reduce cognitive resources available for such internal simulations, perhaps participants in the load condition were less likely to engage in covert imitation of the speech they heard compared to those in the no-load condition. While the current study did not directly measure covert imitation, previous research suggests that resource-demanding conditions may interfere with internal motor-based predictions of speech^2,7^. Future studies could further investigate this possibility by incorporating measures of covert imitation or articulatory simulation.More importantly, our findings indicate that verbal WM load attenuated the growth rate of anticipatory fixations more compared to visual WM load. This suggests a greater disruption to language prediction due to verbal WM load than visual WM load. Notably, prior research demonstrated that these two types of WM load manipulations do not differ significantly in the level of cognitive demand they impose on participants^20^. Moreover, our results showed no significant difference in how verbal and visual WM loads affected participants’ language comprehension performance, further suggesting that any observed effects were not simply due to disparities in task difficulty but rather to the distinct cognitive mechanisms underlying these two types of working memory. Therefore, the stronger disruptive effect of verbal WM load compared to visual WM load suggests that interference with visual processes is not the primary mechanism by which WM load influences predictive fixations. If it were, we would expect predictive fixations to be more disturbed in the visual WM condition. Therefore, we tend to believe that participants predicted upcoming linguistic information through implicit imitation of the speech they heard, and the verbal WM task further occupied the production system due to the need to remember words via phonological rehearsal^47,48^. This greater interference may explain why verbal WM load has a more pronounced effect on language prediction compared to visual WM load.

However, while our findings suggest that the effects observed during the tone sandhi window are primarily phonological (specifically tonal) in nature, the modulation of this phonological prediction by visual and verbal WM load conditions presents a complex pattern. Our cluster-based permutation analysis revealed an earlier onset of increased target fixations in the visual WM group (75 ms post-classifier onset) compared to the verbal WM group (325 ms post-onset). Considering that the latency of eye movements is estimated to be approximately 200 ms in some studies^49^, this might imply a delayed phonological prediction effect under visual working memory load, whereas verbal working memory load potentially eliminated this effect altogether. Nevertheless, given alternative evidence suggesting shorter eye movement latencies (≤ 100 ms) within the visual world paradigm^50^, an alternative interpretation is that both visual and verbal working memory load conditions may have entirely disrupted phonological prediction. This interpretational ambiguity necessitates further investigation in future research to establish more robust conclusions regarding the influence of working memory load modality on phonological prediction.

It is also worth noting that the between-subject design used in this study carries the potential for individual differences to confound the interpretation of the observed effects. While we measured spatial and verbal working memory, and found no significant group differences, we acknowledge these assessments may not fully capture individual variability. Additionally, the relatively small sample size of 24 participants per group might limit the generalizability of our conclusions. Future research endeavors should explore within-subject designs as a means of more rigorously controlling for individual variability, though care must be taken to mitigate potential carryover or interference effects inherent in such designs.

## Conclusion

Employing a dual-task paradigm with eye-tracking in a visual world setting, this study revealed listeners’ anticipatory gaze towards targets, evidencing linguistic prediction. Specifically, early fixations during the tonal cue window indicated prediction of tone in spoken language processing. Moreover, working memory load significantly disrupted these predictive effects, highlighting WM’s role in anticipation. Notably, verbal memory load interfered with language prediction more severely than visual memory load, suggesting that different WM subtypes differentially influence this process, potentially due to the specific cognitive components each engages.

## Data Availability

The datasets, materials and analysis code generated during the current study are available in the OSF repository at https://osf.io/58q9b/?view_only=fe9f7f33a08 d4f72811ae1efd5 d1ac1b.
